# Feasibility of whole‐body MRI for cancer screening in children and young people with ataxia telangiectasia: A mixed methods cross‐sectional study

**DOI:** 10.1002/cam4.70049

**Published:** 2024-07-26

**Authors:** Renata Neves, Rafal Panek, Katie Clarkson, Ouliana Panagioti, Natasha Schneider Fernandez, Sophie Wilne, Mohnish Suri, William P. Whitehouse, Sumit Jagani, Madhumita Dandapani, Cris Glazebrook, Robert A. Dineen

**Affiliations:** ^1^ Radiological Sciences, Mental Health and Clinical Neuroscience, School of Medicine University of Nottingham Nottingham UK; ^2^ Department of Radiology Nottingham University Hospitals NHS Trust Nottingham UK; ^3^ Medical Physics and Clinical Engineering Nottingham University Hospitals NHS Trust Nottingham UK; ^4^ School of Medicine University of Nottingham Nottingham UK; ^5^ School of Sport, Exercise and Health Sciences Loughborough University Loughborough UK; ^6^ NIHR Nottingham Clinical Research Facility Nottingham UK; ^7^ Independent Patient and Parent Representative, c/o Radiological Sciences, Mental Heatlh and Clinical Neuroscience, School of Medicine University of Nottingham Nottingham UK; ^8^ Department of Paediatric Oncology Nottingham University Hospitals NHS Trust Nottingham UK; ^9^ Nottingham Clinical Genetics Service Nottingham University Hospitals NHS Trust Nottingham UK; ^10^ Paediatric Neurology Nottingham University Hospitals NHS Trust Nottingham UK; ^11^ Department of Radiology, Nottingham Children's Hospital Nottingham University Hospitals NHS Trust Nottingham UK; ^12^ Children's Brain Tumour Research Centre University of Nottingham Nottingham UK; ^13^ Institute of Mental Health University of Nottingham Nottingham UK; ^14^ NIHR Nottingham Biomedical Research Centre Nottingham UK; ^15^ Sir Peter Mansfield Imaging Centre University of Nottingham Nottingham UK

**Keywords:** ataxia‐telangiectasia, cancer predisposition, cancer screening, mixed methods research, psychosocial impact, whole‐body MRI

## Abstract

**Background/Objectives:**

Ataxia telangiectasia (A‐T) is an inherited multisystem disorder with increased sensitivity to ionising radiation and elevated cancer risk. Although other cancer predisposition syndromes have established cancer screening protocols, evidence‐based guidelines for cancer screening in A‐T are lacking. This study sought to assess feasibility of a cancer screening protocol based on whole‐body MRI (WB‐MRI) in children and young people with A‐T.

**Design/Methods:**

Children and young people with A‐T were invited to undergo a one‐off non‐sedated 3‐Tesla WB‐MRI. Completion rate of WB‐MRI was recorded and diagnostic image quality assessed by two experienced radiologists, with pre‐specified success thresholds for scan completion of >50% participants and image quality between acceptable to excellent in 65% participants. Positive imaging findings were classified according to the ONCO‐RADS system. Post‐participation interviews were performed with recruited families to assess the experience of participating and feelings about waiting for, and communication of, the findings of the scan.

**Results:**

Forty‐six children and young people with A‐T were identified, of which 36 were eligible to participate, 18 were recruited and 16 underwent WB‐MRI. Nineteen parents participated in interviews. Fifteen participants (83%) completed the full WB‐MRI scan protocol. The pre‐specified image quality criterion was achieved with diagnostic images obtained in at least 93% of each MRI sequence. Non‐malignant scan findings were present in 4 (25%) participants. Six themes were identified from the interviews: (1) anxiety is a familiar feeling, (2) the process of MRI scanning is challenging for some children and families, (3) preparation is essential to reduce stress, (4) WB‐MRI provides the reassurance about the physical health that families need, (5) WB‐MRI experience turned out to be a positive experience and (6) WB‐MRI allows families to be proactive.

**Conclusion:**

This study shows that WB‐MRI for cancer screening is feasible and well‐accepted by children and young people with A‐T and their families.

## Graphical abstract

Ataxia telangiectasia (A‐T) is an inherited multisystem disorder with increased sensitivity to ionising radiation and elevated cancer risk. Despite this, evidence‐based guidelines for cancer screening in A‐T are lacking. This study sought to assess the feasibility of a cancer screening protocol based on whole‐body MRI (WB‐MRI) in children and young people with A‐T. Our results suggest that WB‐MRI for cancer screening in A‐T is feasible and well‐accepted by children and young people with A‐T and their families.

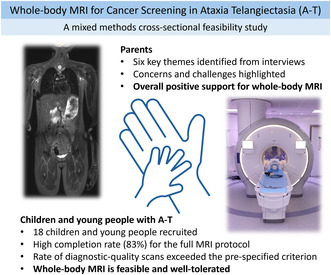

## 1 INTRODUCTION

Ataxia Telangiectasia (A‐T) is a rare autosomal recessive disorder characterised by neurodegeneration, immunodeficiency, respiratory disease, radiosensitivity and cancer susceptibility (14%–24% cumulative incidence in childhood).^1^, ^2^, ^3^, ^4^, ^5^, ^6^ This complex disorder results from mutations in the *ATM* (*ataxia telangiectasia mutated*) gene, which can lead to absent, non‐functioning or hypo‐functioning ATM protein, resulting in genomic instability.^1^, ^2^, ^7^, ^8^ Mutations leading to complete absence of functioning ATM protein result in classical A‐T, with early childhood onset, severe phenotype and death typically in the third decade, whereas mutations allowing residual ATM function result in a milder phenotype referred to as variant A‐T.^9^ The main causes of death in A‐T are cancer and lung disease.^8^, ^10^ Cancers occurring in A‐T include lymphoid malignancies such as lymphoma and leukaemia which dominate in classical A‐T, and solid tumours including brain, breast, gastrointestinal and thyroid malignancies and hepatocellular carcinoma, which are more common in variant A‐T.^3^, ^4^, ^8^, ^11^

The report of the 2016 American Association for Cancer Research Childhood Cancer Predisposition Workshop states that ‘Evidence‐based standards for cancer screening do not exist for patients with A‐T, particularly in childhood’, but recommended consideration of annual physical exam, complete blood count and metabolic profile including lactate dehydrogenase.^12^ In 2017 Van Os and colleagues recommended periodic screening for malignancies in A‐T, including annual blood testing and imaging screening in adults, specifically annual abdominal ultrasound and breast MRI over the age of 25 years.^5^ While helpful, this guidance lacks a firm evidence‐base and does not include recommendations for imaging in children.^5^ A recent international Delphi study of clinical A‐T experts has again highlighted the need for evidence‐based cancer screening guidelines in A‐T, recommending further research to address key evidence gaps for informing guideline development.^13^

Clinical cancer screening guidelines have been developed and implemented for other cancer predisposition syndromes (CPSs) such as Li Fraumeni syndrome/TP53 germline mutation carriers (LFS) and constitutional mismatch repair deficiency syndrome (CMMRD), and have potential to reduce diagnostic delays, detect malignancies at an earlier stage, allow earlier initiation of treatment and improve cancer‐related outcomes.^14^, ^15^, ^16^, ^17^, ^18^, ^19^, ^20^, ^21^, ^22^, ^23^, ^24^ An important feature of these protocols is the use of whole‐body magnetic resonance imaging (WB‐MRI) which provides whole‐body imaging coverage with excellent soft‐tissue contrast resolution for lesion detection without exposure to ionising radiation. WB‐MRI protocols typically include sequences optimised for anatomic detail (e.g. T1‐weighted sequences) as well as pathology detection (e.g. diffusion‐weighted imaging, DWI and STIR), and are increasingly used for detection of both primary and secondary malignancies.^25^, ^26^, ^27^, ^28^, ^29^, ^30^ However, WB‐MRI has limitations including susceptibility to artefacts from patient motion, relatively long imaging times and high rate of incidental, indeterminate and false‐positive findings.^31^, ^32^

While WB‐MRI could be included in future cancer screening guidelines for people with A‐T, the efficacy of this approach should be established in a prospective trial. However, prior to an efficacy trial it is important to establish the technical feasibility of cancer screening with WB‐MRI in a paediatric A‐T population, as this cannot be assumed. Children with A‐T experience involuntary movements and breathing difficulties relating to respiratory problems that could limit the success of image acquisition, and there may be a high rate of false positive findings due to the increased risk of infections and granulomatous disease in A‐T.^8^ In addition to technical aspects and diagnostic performance, the psychosocial impact associated with WB‐MRI screening needs to be considered. As well as the physical and emotional impact of scanning for children, screening can increase anxiety for parents/carers due to the possibility of finding cancer or other pathologies.^26^ A recent qualitative study with people with A‐T and family members found that they want cancer screening because of the potential benefits of early cancer diagnosis, but identified concerns that screening could heighten anxiety related to the scan procedure and potential for an unexpected cancer diagnosis.^33^

The primary aim of this prospective, mixed methods study was to establish the technical feasibility of WB‐MRI for cancer screening in children and young people with A‐T. Secondary aims were to establish the prevalence and spectrum of cancer and non‐cancer abnormalities detected on WB‐MRI in this population and to assess the views of, and psychological impact on, participants and families in response to participating in a feasibility study of WB‐MRI for cancer screening.

## 2 METHODS

### 2.1 Participants

Participants were children and young people with A‐T (diagnosed by genetic testing confirming ATM mutation, plus ATM assay confirming absent, non‐functioning or hypo‐functioning ATM protein kinase) aged 4–18 years recruited according to the eligibility criteria in Table 1, and their parents/guardian. Participants were recruited via the National Paediatric A‐T Clinic held at the Nottingham University Hospitals NHS Trust. The first approach was made by a staff member of the A‐T clinic, who informed the family about the study. Families who expressed interest in the study were provided with an information pack, which included the invitation letter, an age‐appropriate participant information sheet, and a parent/guardian information sheet.

**TABLE 1:** Eligibility criteria for participants with A‐T.

Inclusion criteria	Exclusion criteria
Confirmed diagnosis of A‐T	Contra‐indication to MRI scan
Aged 4–18 years	
Able to undergo MRI scan without sedation or general anaesthetic, after age‐appropriate preparation	
Able to give informed consent (if 16 or older), or have a parent or guardian who is able to give informed consent	

Parental informed consent for all participants aged 15 years and under and informed consent for participants aged 16 years and above was obtained prior to participation. Informed consent was also obtained from the parent/guardian for their participation in the follow‐up interview following their child's WB‐MRI scan. The study underwent research ethics committee review and approval by the UK Health Research Authority (ref. 22/YH/0053). The study was prospectively registered at clinicaltrials.gov (NCT05252819) and the protocol published in an open‐access online research repository prior to commencement of data analysis.^34^

### 2.2 Procedures

#### 2.2.1 Participant preparation

In addition to age‐appropriate participant information sheets, parents/guardians were sent the link to an internet‐based MRI animation prior to the WB‐MRI scan appointment to watch with their child to help prepare them for the scan.^35^ On the day of the scan, age‐appropriate preparation was provided to child participants by an experienced paediatric MRI radiographer (RN), including play preparation and use of an MRI simulator (‘mock’ MRI) or a Philips miniature ‘Kitten Scanner’^36^, ^37^ as appropriate.

#### 2.2.2 The WB‐MRI procedure and clinical reporting

The WB‐MRI was performed using a 3 T Philips Elition MRI system (Philips, Eindhoven, NL) using a 15‐channel head coil and two 32‐channel torso coils. The MRI system included the Philips *Ambient Experience* with in‐built audiovisual entertainment^38^ to improve the participant experience and provide distraction during the MRI scan. A parent was allowed to accompany their child in the scan room during the scan, following mandatory MRI safety screening.^39^

The WB‐MRI protocol included whole‐body axial T1‐weighted mDIXON, axial DWI brain and axial DWIBS, coronal Short Tau Inversion Recovery (STIR) covering torso and neck, sagittal T_1_‐weighted whole‐spine and axial T_2_‐weighted and 3D T_1_‐weighted brain imaging. A summary of the MRI protocol is shown in Table 2 and full details of the scan acquisition parameters are provided in the supplementary file S1.

**TABLE 2:** Summary of whole‐body MRI protocol (full protocol available in supplementary file S1).

WB‐MRI protocol	Anatomic coverage	Imaging stations	Imaging acquisition	Multiplanar reformats
T_1_‐weighted mDixon	Feet to vertex	5–7 (depending on participant height)	Axial	Coronal
DWI and DWIBS	Knee to vertex	4–6 (depending on participant height)	Axial	Coronal 3D‐rotational MIP images
STIR	Torso to neck	2	Coronal	N/A
T_1_‐weighted	Spine	2	Sagittal	N/A
T_2_‐weighted	Brain	1	Axial	N/A
3DT_1_‐weighted	Brain	1	Sagittal	N/A

Abbreviations: MIP, maximum intensity projection; N/A, not applicable.

The MRI scans underwent formal clinical reporting by consultant paediatric radiologist (SJ) and neuroradiologist (RD) who reported the body and brain imaging respectively. The clinical MRI report was sent to the lead clinician at the National Paediatric A‐T Clinic (MS) who communicated results the families and other clinicians caring for the child/young person with A‐T as appropriate, including a recommendation for further investigations when appropriate. When the scan had positive findings the parents were phoned directly by the lead clinician who explained results, which was followed up by a letter detailing the results and next steps. Scan results without positive findings were communicated to parent by letter.

#### 2.2.3 Post‐scan evaluation of participant experience

Immediately following the MRI scan, the children/young people were asked to rate their comfort and experience during the MRI scan using an age‐appropriated 4 Likert scale questionnaire (Supplementary file S2). After the scan results had been communicated to the families or after further examinations were conducted, when required, the parents/guardians and children/young people with A‐T (if they wished) were invited to have a semi‐structured interview (supplementary file S3) to explore the participant's perceptions of the preparation and experience of scanning, and the emotional impacts of awaiting and receiving the findings from the scan. These semi‐structured interviews were conducted on Microsoft Teams, recorded and transcribed verbatim using the record and transcribe option available on Microsoft Teams. For those who had abnormalities detected, the interview also assessed the impact of being referred for further investigations. The interviews were not time‐limited but rather led by the engagement of the participants.

### 2.3 Data analysis

To address the primary aim of establishing the technical feasibility of WB‐MRI for cancer screening in children and young people with A‐T we recorded (i) rate of completion of whole‐body MRI protocol, (ii) duration of scan tolerated (if not full protocol) and (iii) diagnostic image quality assessment. The diagnostic image quality assessment was performed on all sequences by two expert raters (RD and SJ) independently and scores averaged. Motion artefacts were rated using a 5‐point scale based on Andre et al,^40^ and overall diagnostic quality of the scan was rated using an ordinal scale of excellent, good, acceptable and non‐diagnostic. Discrepancies between scans where overall diagnostic quality was rated diagnostic or non‐diagnostic were resolved by discussion and consensus. The pre‐specified success thresholds were scan completion rate >50% and image quality between acceptable to excellent in 65% of those completing the scan.

To address the secondary aim of establishing prevalence and spectrum of cancer and non‐cancer abnormalities we recorded number, types and locations of abnormal findings on WBMRI as reported in the formal clinical reports, and classified these using ONCO‐RADS.^41^

To assess the views of, and psychological impact on, participants and families in response to participating in WB‐MRI for cancer screening we (1) report the ratings and free‐text comments regarding the WB‐MRI scan experience from children/young people gathered immediately after the MRI scan, and (2) used thematic analysis as described by Braun and Clarke^42^ to analyse the semi‐structured interviews. Transcripts were read at least three times to identify initial patterns (familiarisation). Computer software NVivo^43^ was used to analyse the data. The codes identified were combined into possible themes, and the relationship between themes was considered (theme development). A thematic map driven by the data was refined by discussion with three members of the research team (RN, RD and CG).

#### 2.3.1 Sample size and statistical analysis

As this was a feasibility study it was not possible to perform a formal power calculation to inform sample size. A recruitment target of 20 people with A‐T and their families/carers to participate was chosen on pragmatic grounds based on our expected recruitment, allowing us to show feasibility of the approach and to gain an understanding of the spectrum of findings on whole body MRI in this population. In view of this small sample size, statistical analysis was limited to descriptive statistics performed using IBM SPSS v26. To assess the inter‐rater agreement between the two expert raters (RD and SJ) in relation to the image quality and motion in the MRI scans, the Weighted Cohen's Kappa was also calculated.

## 3 RESULTS

**FIGURE 1:**
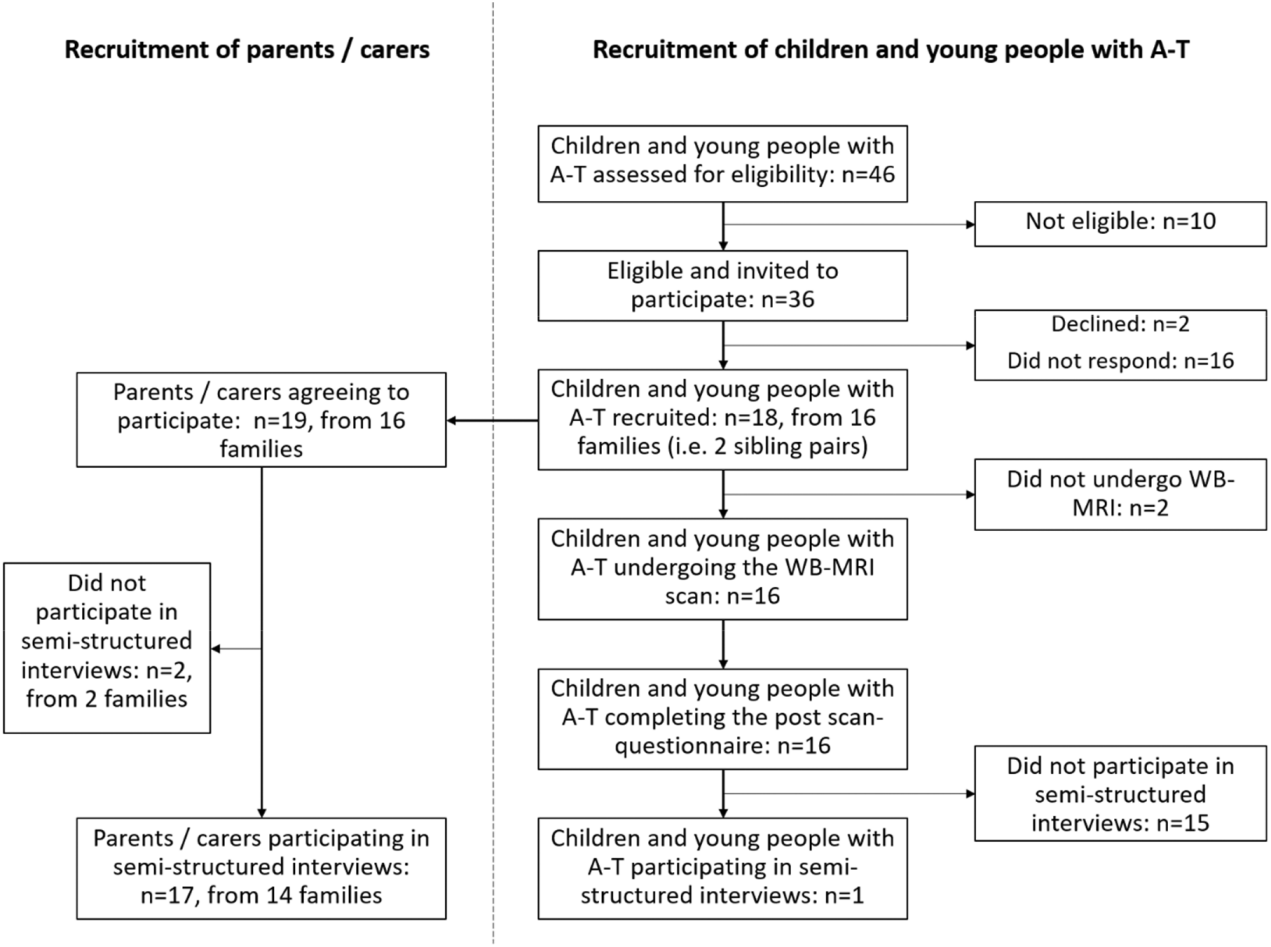
STROBE flowchart to show recruitment.

Recruitment ran from October 2022 to September 2023. Forty‐six children and young people with A‐T were identified, of which 36 were eligible to participate. Ten were excluded (two out of age range, eight with clinician decision not to approach regarding participation due to very recent A‐T diagnosis with family still adjusting to the diagnosis). Eighteen children and young people with A‐T from 16 families were recruited (Figure 1), including two pairs of siblings. Nineteen parents from the 16 families were recruited. In 13 families a single parent consented to participate, and in 3 families both parents consented to participate. Recruitment is shown in Figure 1 and participant demographics are shown in Table 3. The children with A‐T from families who did not respond to the invitation to participate were broadly similar age (median 12 years) and with a similar travelling distance from home to the research centre (mean 114 miles for the recruited families and 116 miles for the non‐responding families).

**TABLE 3:** Participant characteristics.

	Children and young people with A‐T	Parents/carers
Number of participants	18	19
Age in years, median (range)	11, (4–16)	N/A
Sex, male *n* (%)/female *n*, (%)	10 (56%)/8 (44%)	6 (31.6%)/13 (68.4%)
Spoken English, first language *n* (%), second language *n* (%)	10 (55.6%)/8 (44.4%)	11 (57.9)/8 (42.1%)
A‐T subtype, classic *n* (%)/variant *n* (%)	18 (100%)/0 (0%)	N/A
Previous MRI scan, *n* (%) ‐ of which, performed awake *n* (%) ‐ of which, performed with sedation or anaesthesia *n* (%)	13 (72%) 6/13 (46%) 7/13 (54%)	N/A

Abbreviation: N/A, not applicable.

### 3.1 Technical feasibility of WB‐MRI for cancer screening in children and young people with A‐T

Of the 18 children and young people with A‐T recruited, 15 (83.3%) completed the full WB‐MRI, 1 (5.6%, aged 5 years) partially completed the WB‐MRI, and 2 (11.1%, aged 6 and 7 years) declined the scan on the day. Of the 16 participants who had the WB‐MRI, 3 had sequences repeated during the scan due to movement. Median scan duration for participants who completed the WB‐MRI was 53 min (range 39–79 min).

**FIGURE 2:**
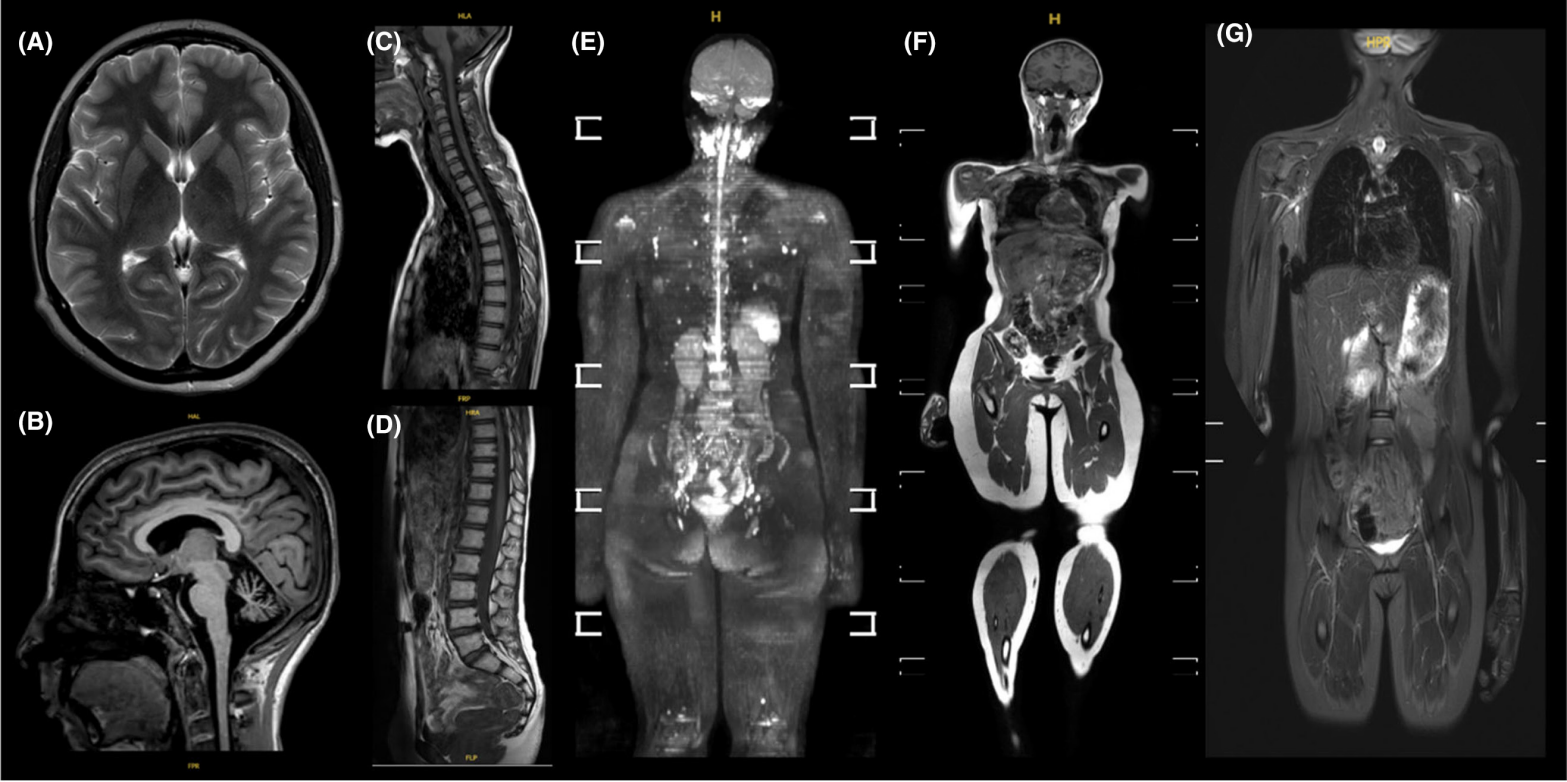
Examples of MRI scans with image quality classified between very good to excellent: (A) axial T2‐weighted brain image; (B) 3D T_1_‐weighted brain image; (C) and (D) sagittal T_1_‐weighted whole‐spine; (E) whole‐body 3D‐rotational MIP b900 DWI; (F) whole‐body coronal T_1_‐weighted mDIXON and (G) whole body coronal STIR.

The pre‐specified success criterion (image quality between acceptable to excellent in 65% of participants completing the scan) was achievable in all the MRI sequences performed, as follows: T_2_‐weighted brain 15/16 (94%), 3D T_1_‐weighted brain 15/16 (94%) DWI brain 15/16 (94%), T_1_‐weighted mDIXON whole‐body 16/16 (100%), DWIBS whole‐body 16/16 (100%), STIR whole‐body 16/16 (100%) T_1_‐weighted spine 14/15 (93%). Example images are shown in Figure 2.

The Weighted Cohen's Kappa showed moderate agreement between the two expert raters (RD and SJ) for image quality assessment (*k* = 0.47, *p* < 0.001) and fair agreement (*k* = 0.31, *p* < 0.001) for motion artefacts.

### 3.2 Prevalence and spectrum of cancer and non‐cancer abnormalities

**FIGURE 3:**
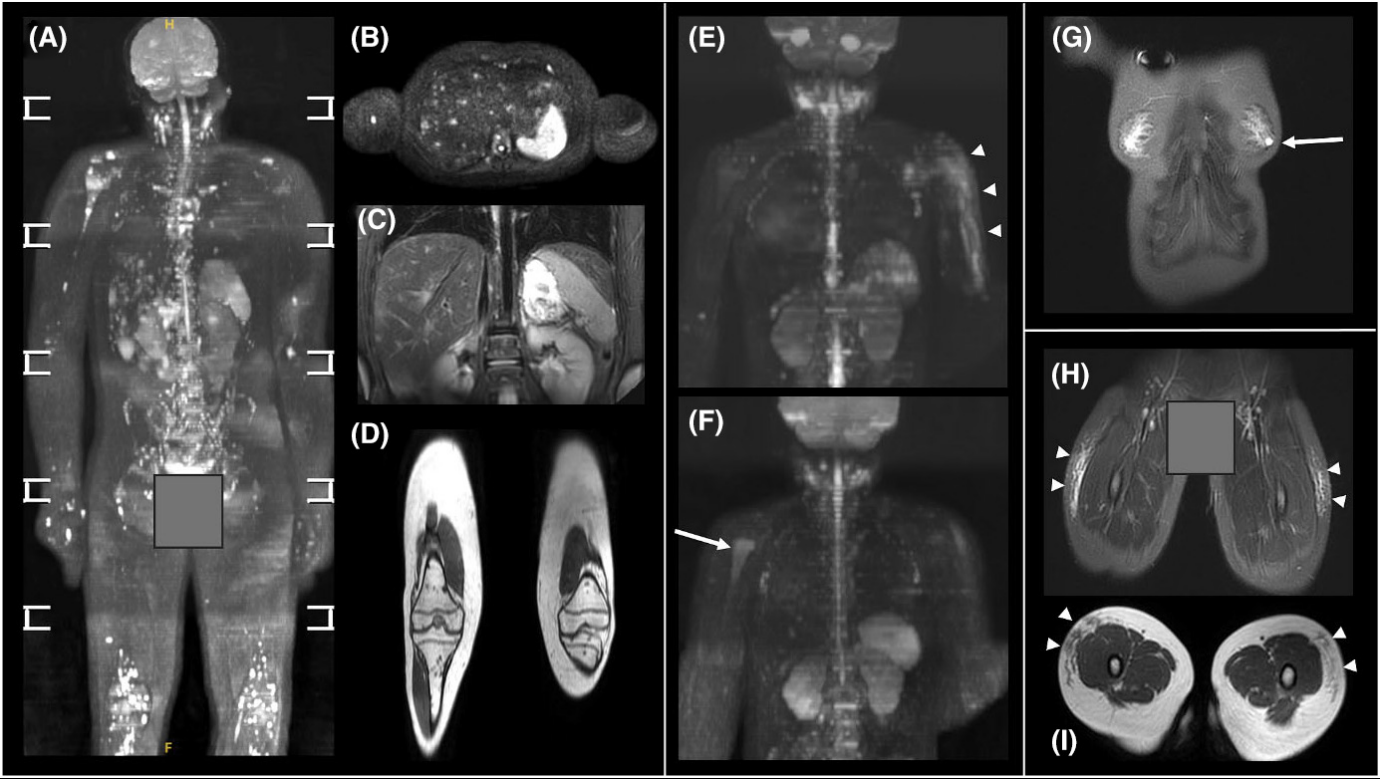
Examples of positive scan findings from four participants: (A) 3D‐rotational MIP b900 DWI image demonstrating multifocal granulomatous disease involving bones, liver and skin; (B) Axial b900 image from the same participant showing the multiple hyperintense liver lesions, as well as lesions in the right humeral shaft and vertebral body; (C) coronal STIR image from the same participant showing multiple hyperintense liver and vertebral body lesons; (D) coronal T_1_‐weighted mDIXON image through the legs of the same participant showing multiple femoral and tibial bone marrow hypontense foci; (E) 3D‐rotational MIP b50 image showing subcutaneous oedema left upper arm (arrow heads); (F) b900 DWI image from the same participant showing high signal right upper humerus (arrow); (G) coronal STIR image showing left sub‐areolar cyst (arrow); (H) coronal STIR and (I) axial T_1_ from a participant with bilateral anterolateral thigh subcutaneous oedema.

Cerebellar atrophy characteristic of A‐T was observed in all participants. Multiple findings were detected in four participants (25%) and were classified according to the ONCO‐RADS system (Table 4, Figure 3). Of these, three participants (18.8%) were referred for further examinations.

**TABLE 4:** Summary of WB‐MRI findings classified according to the ONCO‐RADS category for the four participants with positive scans.

Age (years)^a^	Finding	Sequences finding seen on	ONCO‐RADS classification	Further investigation recommended	Final diagnosis (if known)
13–15	Multiple lesions in liver/spleen skin/and bone lesions (Figure 3A)	STIR/DWIBS/T_1_‐weighted mDIXON	3	Yes—contrast enhanced MRI followed by biopsy of liver and bone lesions	Multiple cutaneous, visceral and bone granulomas
10–12	(1) Small cystic lesion in left breast (Figure 3B)	STIR/DWIBS	2	Breast ultrasound	Benign cyst
	(2) Prominent left sub‐clavicular lymph node	STIR/DWIBS	2	Neck ultrasound	Benign lymph node
4–6	(1) Subcutaneous tissue oedema on the anterolateral thigh (Figure 3C)	STIR/DWIBS	2	No (resolved by clinical discussion)	Immunoglobulin injection site
	(2) Subcutaneous nodules right elbow	STIR/DWIBS	2	No (resolved by clinical discussion)	Granuloma (already known)
4–6	(1) Left shoulder subcutaneous oedema (Figure 3D)	STIR/DWIBS	2	No (resolved by clinical discussion)	Recent vaccination injection site
	(2) Right humerus abnormal bone marrow findings (Figure 3E)	STIR/DWIBS	3	Yes—Targeted MRI and routine blood tests advised	Follow‐up MRI result pending

^a^ Please note, given the small number of participants with a rare disease, to protect participant anonymity when reporting medically relevant findings the age is provided within 3‐year age bands and participant sex is not provided.

### 3.3 Post‐WB‐MRI ratings and comments regarding the WB‐MRI scan experience

Of the 16 participants with A‐T who had the WB‐MRI scan, all provided ratings and comments regarding the MRI experience immediately following the scan (Table 5). Ten (63%) admitted being nervous and worried before the scan and 10 (63%) found it difficult to stay still during the scan. All participants who had the scan said they felt comfortable during the scan and all would be happy to have another MRI scan.

**TABLE 5:** Children's post‐scan questionnaire ratings (*n* = 16).

	Strongly disagree	Disagree	Agree	Strongly agree
Before the scan, participant was not worried about having it	2 (12.5%)	8 (50%)	6 (37.5%)	0
Participant felt comfortable during the scan	0	0	14 (87.5%)	2 (12.5%)
Participant felt nervous during the scan	0	9 (56.3%)	6 (37.5%)	1 (6.3%)
Participant found it easy to stay still during the scan	0	10 (62.5%)	6 (37.5%)	0
Participant found the scan was too noisy	0	10 (62.5%)	6 (3.5%)	0
Participant found that the MRI scan was a nice experience	0	0	12 (75%)	4 (25%)
Participant would be happy to have another scan like this	0	0	10 (62.5%)	6 (37.5%)

### 3.4 Impact of participating in cancer screening using WB‐MRI: Thematic analysis of semi‐structured interviews

Eighteen participants (17 parents and one young person with A‐T aged 12) from 14 families were interviewed in 15 interviews. In two interviews two parents were present. The young person with A‐T was interviewed with the parent present. Two families did not reply to further emails after the child had the MRI scan. Interviews were conducted 3–14 weeks after the WB‐MRI scan. Median interviews duration was 22 min (range 8–44 min).

**FIGURE 4:**
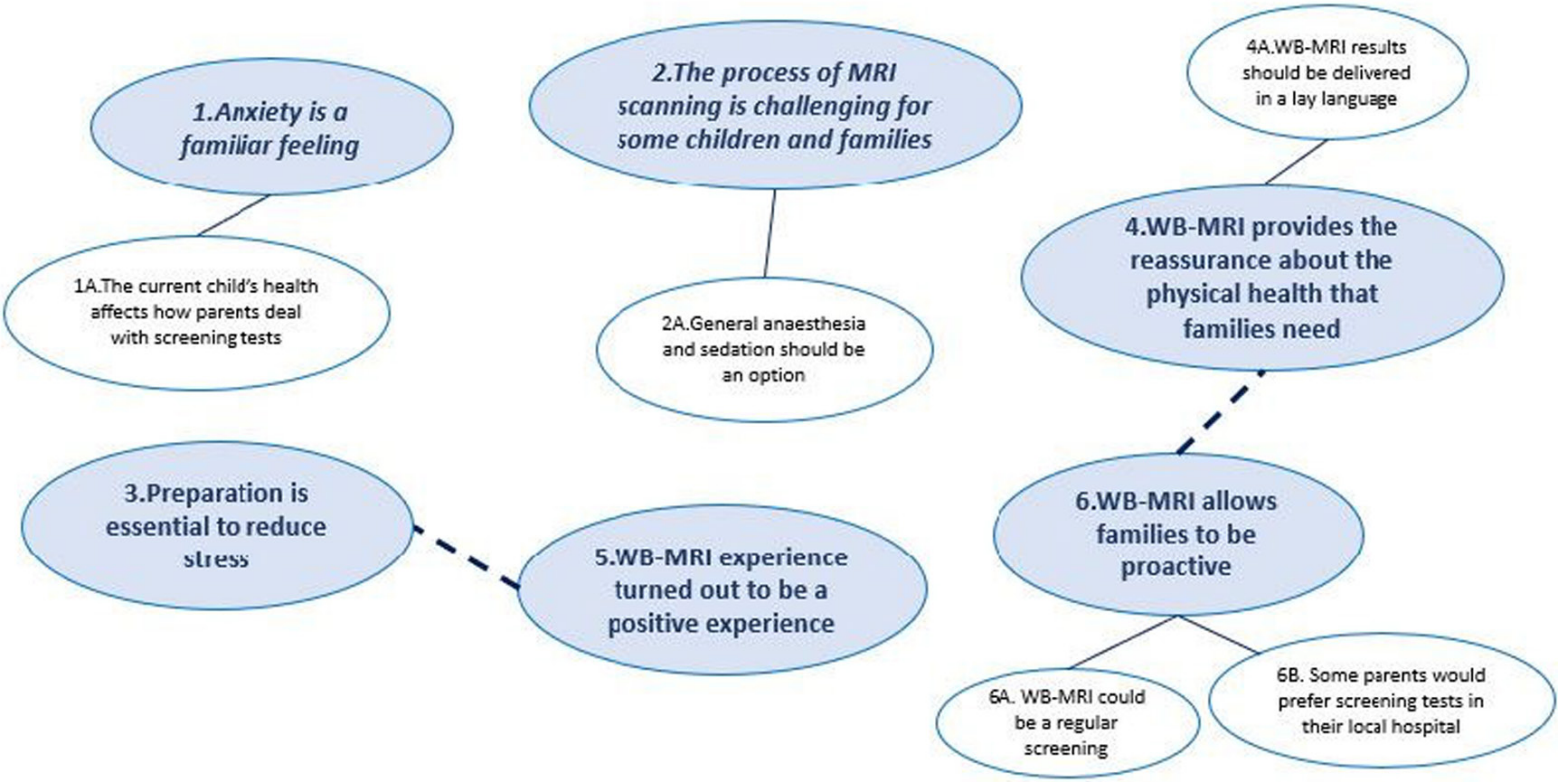
Thematic map illustrating the six main themes (bold font) with the associated subthemes.

Six themes with a total of five subthemes were identified from the data collected (Figure 4). The themes with all illustrative quotes are presented in the supplementary file S4.

#### 3.4.1 Theme 1: Anxiety is a familiar feeling

Overall, parents described anxiety as a normal and pervasive feeling associated with caring for a child with A‐T due to the uncertainty, fear and the worry felt constantly (quote#1). Although screening was associated with an acceptable increased level of worry (quote#2), especially while waiting for the results (quote#3), this seemed to be manageable in the context of the existing fears (quote#4).

So there is no minute in my life that I'm not thinking about it [cancer or other pathologies] and not worrying. You know what's gonna happen? What's ohh, how it's gonna be like and things like that. (…) I'm constantly worried about everything. (Parent_09) quote#1

#### 3.4.2 Subtheme 1A: The child's current health affects how parents deal with screening tests

Parents explained that the current health of their child affected how they saw the screening tests and dealt with the waiting period to get the results. In particular, if the child did not present any concerning symptoms, they tended not to worry as much (quote#5).

I haven't been noticing anything different about them. Like if perhaps would have noticed that they were showing more weakness or fatigue or pale, you know, anything that seemed a bit weird, going into the scan, maybe I would have been more worried. (Parent_01) quote#5

#### 3.4.3 Theme 2: The process of WB‐MRI scanning is challenging for some children and families

Some parents mentioned that they, as well their child, were nervous before the WB‐MRI scan, perhaps due to the anticipation of the unfamiliar (quote#6). They explained that it can be a claustrophobic machine that requires the use of extra equipment, which can increase the feeling of being locked in (quote#7). Some parents emphasised that the mental age of the child can determine the success of the scan (quote#8).

We did have tears, we did have fear you know, we did have all these things, but we still managed to get him in and have the process done. (…) it was the anticipation for, you know, being locked in, you do put a lot on them though. (Parent_013) quote#6

Some parents felt that the WB‐MRI scan was too long, suggesting breaking it down into sections so it could be done in separate appointments (quote#9), especially when the child has involuntary movements, which makes it difficult to stay still for the full duration of the scan (quote#10). Although parents and their children were surprised with how noisy the WB‐MRI scan can be, this did not seem to affect the tolerance of the scan (quote#11).

If the time goes down into sections…I mean even if it's brain one month and then or one year, and then body…if it was like, you know, half an hour there and half an hour here. By splitting for like you know, between like six months or just the timing, yeah, it should be much easier. (Parent_14) quote#9

#### 3.4.4 Subtheme 2A: General anaesthesia and sedation should be an option

Some parents discussed the importance of having general anaesthesia and sedation as an option for the WB‐MRI scan, particularly when the child cannot cope with the scan (quote#12).

Is there a possibility of having that anaesthesia before so he can actually be asleep during the whole process? (Parent_014) quote#12

#### 3.4.5 Theme 3: Preparation is essential to reduce stress

Parents reflected on the preparation given to the child for the WB‐MRI scan. They mentioned the conversations they had which depended on the age of the child (quote#13). When asked about the preparation given by the staff, particularly the YouTube video sent prior to the scan, the parents highlighted how useful they were not only for the child but also for the parents, because they were very informative (quote#14 and quote#15). Parents also emphasised the importance of spending time with the child, explaining all the steps and having age‐appropriate conversations (quote#16 and quote#17). Some parents also discussed the role that a mock scanner can have in preparing a young child to their scan (quote#18).

The whole experience for me was just, you know, being able to be in there with him was huge, that was a big thing (…) the fact, that you're willing to show him things and say that, you know, this isn't going to hurt, this is what it looks like when it goes down. And my head's going to be in there and that sort of stuff. It's more about delivering it softly because they're a child, they're going to get scared (…) I think if you're willing to sit and spend the time with the child, then you can take the fear from it, which is what you did. (Parent_13) quote#16

#### 3.4.6 Theme 4: WB‐MRI provides the reassurance about the physical health that families need

All parents reported that screening tests like WB‐MRI scan provides a sense of reassurance and peace of mind that the families need (quote#19 and quote#20), particularly a whole‐body scan because allows to assess the full body (quote#21).

I have got this peace of mind that, you know, her whole body was checked and it's clear. (Parent_12) quote#21

#### 3.4.7 Subtheme 4A: WB‐MRI results should be delivered in a lay language

Regarding the letter with the WB‐MRI results, parents explained that they had difficulties in understanding what was written due to the language used. They emphasised that this letter should be written in a lay language (quote#22). In fact, some parents mentioned that they would prefer to have the results by phone call rather than in a letter because it allows them to clarify any medical terms they may not understand, however if this is not possible then they suggested to add a contact number in the letter in case they need further clarification (quote#23). This is particularly important when there are findings to be reported, for example, the neurological deterioration typical of A‐T, which was difficult to accept (quote#24).

If we hadn't had the phone call with the professor, how it's written is not, I mean, I can read it and I understand what it's saying. But It doesn't, it's not clear to someone who's not a doctor. Like I am far from a doctor, you know, the wording isn't like, hum, easy to understand. (Parent_04) quote#22

#### 3.4.8 Theme 5: WB‐MRI experience turned out to be a positive experience

Overall, parents reported that the experience of having the WB‐MRI scan and the interaction with the staff was a positive experience. Indeed, they felt welcomed, well‐informed and prepared for the scan (quote#25). They also mentioned that they were happy with how the WB‐MRI scan was performed, more precisely, being able to be with their child during the scan and the constant communication between the staff and the child during the scan (quote#26). Regarding the in‐scanner audio‐visual entertainment, all participants emphasised that this was essential for the success of the scan because it provided a distraction (quote#27). In fact, all parents reported that their child would be happy to have another MRI scan (quote#28), with some mentioning that they would prefer to travel to have the scan in the same facilities (quote#29 and quote#30).

It was a very good experience. And you were, you know, like I said, you were really kind and nice to her and all. And she really liked that. So, she was very happy. She had no complaints. I think it was very good. (Parent_012) quote#25

#### 3.4.9 Theme 6: WB‐MRI allows families to be proactive

Parents highlighted that early detection was one of the main reasons for doing screening tests, such as WB‐MRI scan (quote#31). They emphasised that screening allows them to be proactive in a sense that increases the chances of detecting lesions in the early stages before the child shows symptoms, which could lead to an earlier treatment with better outcome (quote#32). The parents believed that by doing regular screening and monitoring, they were doing their best to provide the best life for their children (quote#33).

Some parents shared that the WB‐MRI scan was the key to initiating further tests. Indeed, they reported that before the WB‐MRI scan, the medical approach adopted was to monitor the child physically. However, with the results of the WB‐MRI, this approach changed drastically, particularly, further tests were recommended for diagnosis purposes (quote#34).

We want him to have the best life that he can. (…) It was more about if they did find something that we could get on and do something preventative, we could get on and catch something early, you know? (Parent_13) quote#33

#### 3.4.10 Subtheme 6A: WB‐MRI could be a regular screening

When discussing the frequency of the WB‐MRI scan for screening purposes, the parents explained that this needs to be decided by the medical team, but the majority felt that doing the WB‐MRI once a year would be acceptable (quote#35).

I'd be happy to do it once every six months, you know, once every year. (Parent_13) quote#35

#### 3.4.11 Subtheme 6B: Some parents would prefer screening tests in their local hospital

Some parents mentioned that they would prefer to have the screening tests done in their local hospitals to avoid travelling long‐distance, which can be complicated for some families (quote#36).

If it was implemented in more hospitals, so there was less of a drive (…) Three hour drive is not fun for anyone. (Parent_07) quote#36

## 4 DISCUSSION

The primary aim of this prospective study was to establish the technical feasibility of WB‐MRI for cancer screening in children and young people with A‐T, and our results indicate that feasibility is confirmed. We found high compliance amongst participating children and young people, with 83% completing the WB‐MRI protocol which exceeded the pre‐specified success criterion of >50%. We also found a high rate of scans achieving adequate (or better) diagnostic image quality (93%) in those completing the WB‐MRI protocol, which exceeded the pre‐specified image quality criterion of >65%.

Although no lesions with high likelihood of malignancy were identified, findings on whole‐body MRI that required further clinical evaluation or imaging investigation were common (25% of participants). These included cutaneous, visceral and osseous granulomas which are known to occur in A‐T, subcutaneous oedema from recent immunoglobulin and vaccination injections, benign cysts and prominent lymph nodes and localised bone marrow signal abnormality. The relatively high rate of findings with low or intermediate likelihood of malignancy needs to be considered for any future screening trial or programme using WB‐MRI for people with A‐T, given the additional medical investigations and associated anxiety that these findings may lead to.^20^, ^44^, ^45^

A secondary aim of the study was to assess the views of, and psychological impact on, children/young people with A‐T and their families in response to participating in a feasibility study of WB‐MRI for cancer screening. The thematic analysis of semi‐structured interviews performed with parents (and one young person with A‐T) after the scan provided rich information on their reflections of the experience. Parents felt positive following the scan, and participation made families feel reassured and more empowered in terms of participating in proactive cancer detection. An overall positive view was also given by the children and young people immediately after the scan, with all saying they would be happy to have another MRI scan which is important for a future screening trial or programs that would require repeated scans. These results align with previous studies that assess the psychosocial impact of cancer screening in people affected with Li‐Fraumeni. They reported high level of acceptability with results suggesting that cancer screening programmes can provide a sense of control and security without adding a significant long‐term psychosocial burden.^44^, ^45^, ^46^, ^47^, ^48^

Parents reported an increase in anxiety in anticipation of the scan and receiving the results, but contextualised this against the fact that living with A‐T is already emotionally demanding and some felt they have developed the resilience needed to manage the additional short‐term anxiety.^33^, ^49^ ‘Scanxiety’ was reported in similar studies related to cancer surveillance and referrers to the temporary increase of anxiety observed around the screening time and during the waiting period to receive the results.^44^, ^45^, ^46^ Nonetheless, appropriate support for people with A‐T and family members to help with screening‐associated anxiety needs careful consideration if future screening trials or programs are developed. Indeed, some studies suggested the use of a screening tool to identify the families who may need individual psychological support or develop a comprehensive care program to provide longitudinal, individualised, age‐appropriate support.^49^, ^50^, ^51^

Challenges associated with MRI scans were highlighted by parents, in particular the length of the scan, the noise, and how scary it can be for young children,^25^, ^52^ and the parents preference to have the scanning performed at a local hospital.^47^ These aspects emphasise the importance of preparation of the child/young person (over half of the participants with A‐T reported feeling nervous before the scan) and also providing clear information and pragmatic support for parents in the run up to the scan. In fact, previous studies demonstrated the effectiveness that preparation techniques, comprehensive information and age‐appropriated communication have in the success rate of performing good quality MRI scans.^35^, ^53^, ^54^, ^55^, ^56^ Nonetheless, shortening the duration of the scan as a result of technical innovations may help with compliance, but for children unable to undergo the scan while awake options for performing WB‐MRI under sedation or anaesthesia could be considered.

The combination of both positive and negative themes identified in the qualitative analysis mirrors previous studies in other CPS which have reported mixed psychosocial outcomes: although anxiety, fear and cancer worry were reported, there was a positive attitude towards screening with higher levels of satisfaction and acceptability.^20^, ^44^, ^45^, ^46^, ^48^, ^57^ Future screening trials or programs of WB‐MRI should include careful monitoring of psychosocial outcomes in addition to cancer‐related detection, morbidity and mortality outcomes.

### 4.1 Limitations

One limitation of this study was its small sample size. A‐T is a rare condition, and we recruited a range of ages across childhood (4–16 years). A larger sample may have revealed other views in relation to the impact of screening, but we believe the risk of this is low given the fact that data saturation^58^ was achieved in our analysis. Another study limitation was the period between the MRI scan and the post‐participation interviews, especially in families who were referred for further tests as per their request. However, we believed that assessing the impact of having additional tests was as important as evaluating the impact of the MRI scan.

Importantly, since this study was conducted at one time point, it is unknown whether the participants would develop screening fatigue in the long‐term. Nevertheless, all the participants expressed the desire to continue screening with some suggesting an ideal interval period between screening tests.

Finally, the views of the families who did not reply to the invite, or who declined to participate were not captured. These families may have given different insights into screening in A‐T, which would be equally important when designing a cancer screening programme. However, it should be noted that the aim of this study was to assess the feasibility of this approach so a large prospective trial could be designed based on the results obtained.

## 5 CONCLUSION

This study finds that WB‐MRI for cancer screening in A‐T is technically feasible and well tolerated by the children and young people undergoing the scan. Cancer screening using WB‐MRI was positively viewed by families although short‐term anxiety and need for clear communication and practical support were highlighted. This study does not provide evidence of the efficacy of the approach for detecting malignancy in people with A‐T but provides a basis on which to develop a prospective efficacy trial.

## AUTHOR CONTRIBUTIONS

**Renata Neves:** Data curation (equal); formal analysis (equal); investigation (equal); methodology (equal); project administration (equal); validation (equal); visualization (equal); writing – original draft (equal); writing – review and editing (equal). **Rafal Panek:** Conceptualization (equal); data curation (equal); funding acquisition (equal); investigation (equal); methodology (equal); validation (equal); writing – review and editing (equal). **Katie Clarkson:** Formal analysis (equal); investigation (equal); writing – review and editing (equal). **Ouliana Panagioti:** Investigation (equal); writing – review and editing (equal). **Natasha Schneider Fernandez:** Writing – review and editing (equal). **Sophie Wilne:** Conceptualization (equal); funding acquisition (equal); methodology (equal); writing – review and editing (equal). **Mohnish Suri:** Conceptualization (equal); funding acquisition (equal); methodology (equal); writing – review and editing (equal). **William P. Whitehouse:** Conceptualization (equal); funding acquisition (equal); methodology (equal); writing – review and editing (equal). **Sumit Jagani:** Conceptualization (equal); formal analysis (equal); funding acquisition (equal); methodology (equal); writing – review and editing (equal). **Madhumita Dandapani:** Conceptualization (equal); funding acquisition (equal); methodology (equal); writing – review and editing (equal). **Cris Glazebrook:** Conceptualization (equal); funding acquisition (equal); methodology (equal); supervision (equal); writing – review and editing (equal). **Robert A. Dineen:** Conceptualization (equal); data curation (equal); formal analysis (equal); funding acquisition (equal); methodology (equal); project administration (equal); supervision (equal); validation (equal); visualization (equal); writing – review and editing (equal).

## FUNDING INFORMATION

This study is funded by a grant from Action for A‐T (ref. 20NOT05/David Peake Fund). Renata Neves is supported by a Doctoral Fellowship awarded by the College of Radiographers (ref. DF021). This study is supported by the National Institute for Health Research (NIHR) Applied Research Collaboration East Midlands (ARC EM). The views expressed are those of the author(s) and not necessarily those of the NIHR or the Department of Health and Social Care.

## CONFLICT OF INTEREST STATEMENT

The authors declare no conflict of interest.

## PATIENT OR PUBLIC CONTRIBUTION

An independent parent representative contributed to the study, supporting the research team in interpreting and commenting on the appropriateness of the language used in this report.

## Supporting information


Data S1:

## Data Availability

The data that support the findings of this study are available from the corresponding author upon reasonable request.
