# Supplementary material for: Feasibility of whole‐body MRI for cancer screening in children and young people with ataxia telangiectasia: A mixed methods cross‐sectional study
Source: Cancer Med. 2024 Jul 26;13(14):e70049. doi: 10.1002/cam4.70049 (PMC11273546; doi:10.1002/cam4.70049)
Supplement: Supplementary file 1 — Data S1: [file CAM4-13-e70049-s001.zip › WB5AE6~1.DOC]

**Feasibility of whole-body MRI for cancer screening in children and young people with Ataxia Telangiectasia: a mixed methods cross-sectional study**

***Supplementary file 2* Post-scan questionnaire**

These questions are to assess the experience of the young person who had the MRI scan


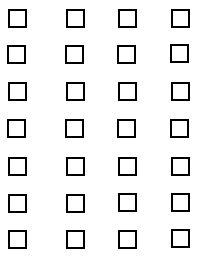
Participant ID __________________

Strongly Strongly

Disagree Disagree Agree Agree

|  |
| --- |
| 1. Before the scan, the participant was not worried about having it |
| 1. The participant felt comfortable during the scan |
| 1. The participant felt nervous during the scan |
| 1. The participant found it easy to stay still during the scan |
| 1. The participant found the scan was too noisy |
| 1. The participant found that the MRI scan was a nice experience |
| 1. The participant would be happy to have another scan like this |
| 1. Extra comments made by the participant 2. Has the participant had an MRI scan before?   9.1 If so, was it under general anaesthesia? |
|  |


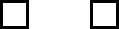

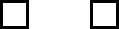


Yes No
