# Supplementary material for: Feasibility of whole‐body MRI for cancer screening in children and young people with ataxia telangiectasia: A mixed methods cross‐sectional study
Source: Cancer Med. 2024 Jul 26;13(14):e70049. doi: 10.1002/cam4.70049 (PMC11273546; doi:10.1002/cam4.70049)
Supplement: Supplementary file 1 — Data S1: [file CAM4-13-e70049-s001.zip › WBMRIF~2.DOC]

**Feasibility of whole-body MRI for cancer screening in children and young people with Ataxia Telangiectasia: a mixed methods cross-sectional study**

***Supplementary file 4* Themes and subthemes with illustrative quotes**

| **Themes and subthemes** | **Quotes** |
| --- | --- |
| ***Theme 1***  **Anxiety is a familiar feeling** | “*So there is no minute in my life that I'm not thinking about it and not worrying. You know what's gonna happen? What's ohh, how it's gonna be like and things like that. (…) I'm constantly worried about everything.” (Parent_09) quote#1*  *“But I think there is always in any hospital appointments, unfortunately, because of the nature of the condition, there is anxiety around it****.”*** *(Parent_02) quote#2*  *“It [the waiting period for the results] was alright. We just, we are used to it…” (Parent_011) quote#3*  *“Even though there was no reason to think [retracted] had anything, waiting for any results, it's horrible” (Parent_07) quote#4* |
| *Subtheme 1A:* The child’s current health affects how parents deal with screening tests | *“I haven't been noticing anything different about them. Like if perhaps would have noticed that they were showing more weakness or fatigue or pale, you know, anything that seemed a bit weird, going into the scan, maybe I would have been more worried” (Parent_01) quote#5* |
| ***Theme 2***  **The process of MRI scanning is challenging for the child and the family** | *“We did have tears, we did have fear you know, we did have all these things, but we still managed to get him in and have the process done. (…) it was the anticipation for, you know, being locked in, you do put a lot on them though.” (Parent_013) quote#6*  *“And she didn't particularly like the tunnel. Like I say, she put up with it” (Parent_02) quote#7*  *“It's just not for kids with his like his mentality and movements.” (Parent_06) quote#8*  *“If the time goes down into sections... I mean even if it's brain one month and then or one year, and then body … if it was like, you know, half an hour there and half an hour here. By splitting for like you know, between like six months or just the timing, yeah, it should be much easier.” (Parent_14) quote#9*  *“It was hard for him to keep perfectly still due to his, you know, twitches and movement.” (Parent_09) quote#10*  *“I only saw his big eyes in the mirror when the first scan started, the eyes were very big...he was surprised at how noisy it was. But yeah, then he could just watch the movie, looking at me.” (Parent_09) quote#11* |
| *Subtheme 2A:* General anaesthesia and sedation should be an option | *“Is there a possibility of having that anaesthesia before so he can actually be asleep during the whole process?“ (Parent_014) quote#12* |
| ***Theme 3***  **MRI provides the reassurance about the physical health that families need** | *“It was reassuring to know that there was nothing that you, the thing you were looking for wasn't there. So that's brilliant.” (Parent_2) quote#13*  *“So that was good news and I think it's just reassuring to know that there was nothing picked up. And if something was picked up, I would also feel grateful for having done it.” (Parent_1) quote#14*  *“I have got this peace of mind that, you know, her whole body was checked and it's clear.” (Parent_12) quote#15* |
| *Subtheme 3A:* MRI results should be delivered in a lay language | *“If we hadn't had the phone call with the professor, how it's written is not, I mean, I can read it and I understand what it's saying. But It doesn't, it's not clear to someone who's not a doctor. Like I am far from a doctor, you know, the wording isn't like, hum, easy to understand” (Parent_04) quote#16*  *“Maybe just to receive a letter is OK, but with the chance of maybe there is a number somewhere that you could call and just to get the reassurance you know?” (Parent_09) quote#17*  *“There was the part about the brain, the results about the cerebellum, which obviously is a bit shocking. But you know, I think I would still want to know, like if they had another scan, I would still want to know where things are.” (Parent_01) quote#18* |
| ***Theme 4***  **Preparation is essential to reduce stress** | *“She [daughter] was ready for the scan. She watched a couple of videos before meeting you for the MRI. Without any stress, without…. She was relaxing. (…) I really tried to explain to her how it's important for her to go ahead with MRI.” (Parent_05) quote#19*  *“Having the little videos that you provided about how children go in to have an MRI and things like that. [retracted] was actually quite excited.” (Parent_02) quote#20*  *“The YouTube videos, hum, which I thought were really, really helpful even just from like a parent's perspective because I didn't really know what to expect” (Parent_04) quote#21*  *“The whole experience for me was just, you know, being able to be in there with him was huge, that was a big thing (…) the fact, that you're willing to show him things and say that, you know, this isn't going to hurt, this is what it looks like when it goes down. And my head's going to be in there and that sort of stuff. It's more about delivering it softly because they're a child, they're going to get scared (…) I think if you're willing to sit and spend the time with the child, then you can take the fear from it, which is what you did.” (Parent_13) quote#22*  *“The two things that are key are, well three things: the TV, having the parent there, and calm and age-appropriate conversations.” (Parent_10) quote#23*  *“I think the little machine you had before for it to play with, that was fantastic because I think that definitely helped her know what was gonna happen. That was brilliant.” (Parent_07) quote#24* |
| ***Theme 5***  **MRI experience turned out to be a positive experience** | *“It was a very good experience. And you were, you know, like I said, you were really kind and nice to her and all. And she really liked that. So she was very happy. She had no complaints. I think it was very good.” (Parent_012) quote#25*  *“It's very important definitely to talk, you know, to talk to a patient like between scans, just to reassure, you know, how long the next scan is gonna be, is going to be noisy, the table will move so they know, not all the sudden movement forward and backward.. Everything was very well explained prior to the scan, during the scan, you gave the buzzer, he was comfortable…” (Parent_09) quote#26*  *“Definitely, the movie's a big thing, especially for children. I do MRI every year from breast surveillance, you know, it's hard work and I have the music, but it just drags and drags for 45 minutes it goes on and on… the anxiety, builds up. So I think the movie really takes all the anxiety away.” (Parent_10) quote#27*  *“We’ve already discussed it that, and we’d be happy to do it again.” (Parent_13) quote#28*  *“He would feel better if it was done around you. And for me, I don't mind travelling. (…) if they don't have that facility at my local hospital [DVD], then you know, dependent on him, I think that he would rather come and see you and do that with TV because you've* ***had*** *that first interaction with him.” (Parent_13) quote#29*  *“I think if somewhere more local didn't have a TV, Nottingham would be preferable for that reason.” (Parent_03) quote#30* |
| ***Theme 6***  **MRI allows families to be proactive** | *“In terms of the opportunities to catch up things that might be there that we don’t want there. Like it would make sense to keep track of these things and ensure that we’re on top of them and ahead of them, hopefully. (…) I’ can’t Imagine what the family is going… Sorry…. [getting upset] What they’re going through when they have a diagnosis like that [cancer diagnosis]. On top of having an A-T diagnosis. And so for me, I’ll try and put it sort of to the back of my mind and actually see it as a necessary screening process. (…) I think MRI scans are a necessary precaution because then I’m doing all that we can for her to try and help catch things maybe early. (Parent_02) quote#31*  *“We want to be proactive. If you found something, we’d wanna know anyway, because we’d wanna be able to, obviously treat anything as quick as possible, It’s given us the possibility of being proactive. She might not show symptoms, but it could still be there.” (Parent_07) quote#32*  *“We want him to have the best life that he can. (…) It was more about if they did find something that we could get on and do something preventative, we could get on and catch something early, you know?” (Parent_13) quote#33*  *“If we didn’t have that MRI, I would be in a worse position. Before the MRI, [retracted] Hospital said ‘we’re just gonna leave it on’, watch it and see approach sound like. So, I said ‘well, what are you waiting for? Are you waiting for something to happen?’. They weren’t willing to do anymore. Now, because of the MRI and because we’ve got the backing of your images and Nottingham, they couldn’t do more for us. So it has changed, before the MRI, it was wait and see approach, now It’s literally every test under the sun they’re doing on him. (…) It completely changed the course of action from completely ignoring me to taking me seriously.” (Parent_10) quote#34* |
| *Subtheme 6A:* MRI could be a regular screening | *“I'd be happy to do it once every six months, you know, once every year.” (Parent_13) quote#35* |
| *Subtheme 6B:* Some parents would prefer screening tests in their local hospital | *“If it was implemented in more hospitals, so there was less of a drive (…) Three hour drive is not fun for anyone.” (Parent_07) quote#36* |

NB: Bold font indicates main themes.
