# Supplementary material for: Feasibility of whole‐body MRI for cancer screening in children and young people with ataxia telangiectasia: A mixed methods cross‐sectional study
Source: Cancer Med. 2024 Jul 26;13(14):e70049. doi: 10.1002/cam4.70049 (PMC11273546; doi:10.1002/cam4.70049)
Supplement: Supplementary file 1 — Data S1: [file CAM4-13-e70049-s001.zip › WBMRIF~3.DOC]

**Feasibility of whole-body MRI for cancer screening in children and young people with Ataxia Telangiectasia: a mixed methods cross-sectional study**

| WB-MRI protocol parameters | T_2_-weighted  Brain | 3DT_1_-weighted  Brain | STIR  Whole-body | DWI  brain | DWIBS  Whole-body | T_1_-weighted mDixon | T_1_-weighted  Spine |
| --- | --- | --- | --- | --- | --- | --- | --- |
| TR | 3000 | 6.5 | 4989 | 5228 | 8020 | 4.1 | 489 |
| TE | 90 | 3.1 | 75 | 61 | 56 | (1)1.34 / (2) 2.6 | 8 |
| Flip angle | 90 | 8 | N/A | 90 | N/A | 15 | 90 |
| b-factors (b-values averages) | N/A | N/A | N/A | b-50 (1)  b-900 (3) | b-50 (2)  b-900 (5) | N/A | N/A |
| Slice thickness (mm) | 4 | 1 | 5 | 2.5 | 5 | 4 | 3.5 |
| Slices | 30 | 160 | 35 | 50 | 40 | 125 | 12 |
| FOV (mm) | 182(RL)x  230(AP)x  149(FH) | 160(RL)x  224(AP)x  224(FH) | 450(RL)x  209(AP)x  450(FH) | 484(RL)x  348(AP)x  174(FH) | 484(RL)x  348(AP)x  239(FH) | 550(RL)x  299(AP)x  250(FH) | 53(RL)x  180(AP)x  360(FH) |
| Acquisition voxel size (mm) | 0.55x0.65x4 | 1x1x1 | 1.5x1.5x5 | 2.5x2.5x2.5 | 3x3x5 | 1.5x1.5x4 | 0.85x1.13x3.5 |
| Sense/ Compressed sensing | N/A | 2 | 2.5 (RL/FH) | 2.5 (AP) | 2 (AP) | 2 (AP)  1 (FH) | 1.5 (FH) |
| NSA | 1 | 1 | 1 | 2 | 1 | 1 | 1 |
| Fat saturation technique | N/A | N/A | N/A | SPIR | SPIR | N/A | N/A |
| Acquisition mode | Cartesian | Cartesian | Multivane | Cartesian | Cartesian | Cartesian | Cartesian |

***Supplementary file 1* Whole-body MRI protocol**

RL=Right-left; AP= Anterior-posterior; FH= Foot-head
