# Supplementary material for: Feasibility of whole‐body MRI for cancer screening in children and young people with ataxia telangiectasia: A mixed methods cross‐sectional study
Source: Cancer Med. 2024 Jul 26;13(14):e70049. doi: 10.1002/cam4.70049 (PMC11273546; doi:10.1002/cam4.70049)
Supplement: Supplementary file 1 — Data S1: [file CAM4-13-e70049-s001.zip › WBMRIF~4.DOC]

**Feasibility of whole-body MRI for cancer screening in children and young people with Ataxia Telangiectasia: a mixed methods cross-sectional study**

***Supplementary file 3* Interview schedule**

*Please note: This is a semi-structured interview schedule designed to be used flexibly by the interviewer with each participant/focus group. Therefore, questions (numbered) and prompts (lettered) used per participant/focus group may vary slightly.*

1. Please can you tell me/us about your experience of being involved in the study?

1. What did you and your child think of the MRI and study procedures?
   1. Was the MRI scan explained well to you and your child? If they received preparation e.g., with the play specialist, do you think this was adequate?
   2. Was there anything else that might have helped to make you or your child more comfortable? How could we improve the MRI experience?
   3. Would you be happy to have another MRI scan?
   4. If your child had a blood test, how was it? Would your child agree to having another?
   5. Have your thoughts and feelings about the study procedures (including MRI and the blood test) changed following your/your child’s participation?
2. What impact did your child experience as a result of participating in this trial? Any emotional impacts?
   1. What about you? How did you feel?
   2. What about other members of the family? How did they feel?
   3. How did you feel whilst you were waiting for the results of the scan and the blood test, if this was done?
3. Did you have concerns about the possibility of a positive diagnosis of cancer (i.e., the need for further tests and treatments)?
   1. What were the particular concerns?
   2. Were these concerns sufficient to make you consider whether taking part in a cancer screening programme is worthwhile?
4. Now that you and your child have participated, how often would be too often for you / your family, in terms of attending your local Children’s Hospital for cancer screening scans?
   1. For example, would once a year be acceptable? How about if every 6 months was the recommendation?
5. How do you think we could improve the surveillance programme in the future?

1. Would you recommend this surveillance programme to other children/guardians of children affected by A-T?
   1. Do you think it is worth investigating further in an international trial?

1. Is there anything else you would like to talk to me/us about?
