# Supplementary figures and images for: Feasibility of whole‐body MRI for cancer screening in children and young people with ataxia telangiectasia: A mixed methods cross‐sectional study
Source: Cancer Med. 2024 Jul 26;13(14):e70049. doi: 10.1002/cam4.70049 (PMC11273546; doi:10.1002/cam4.70049)

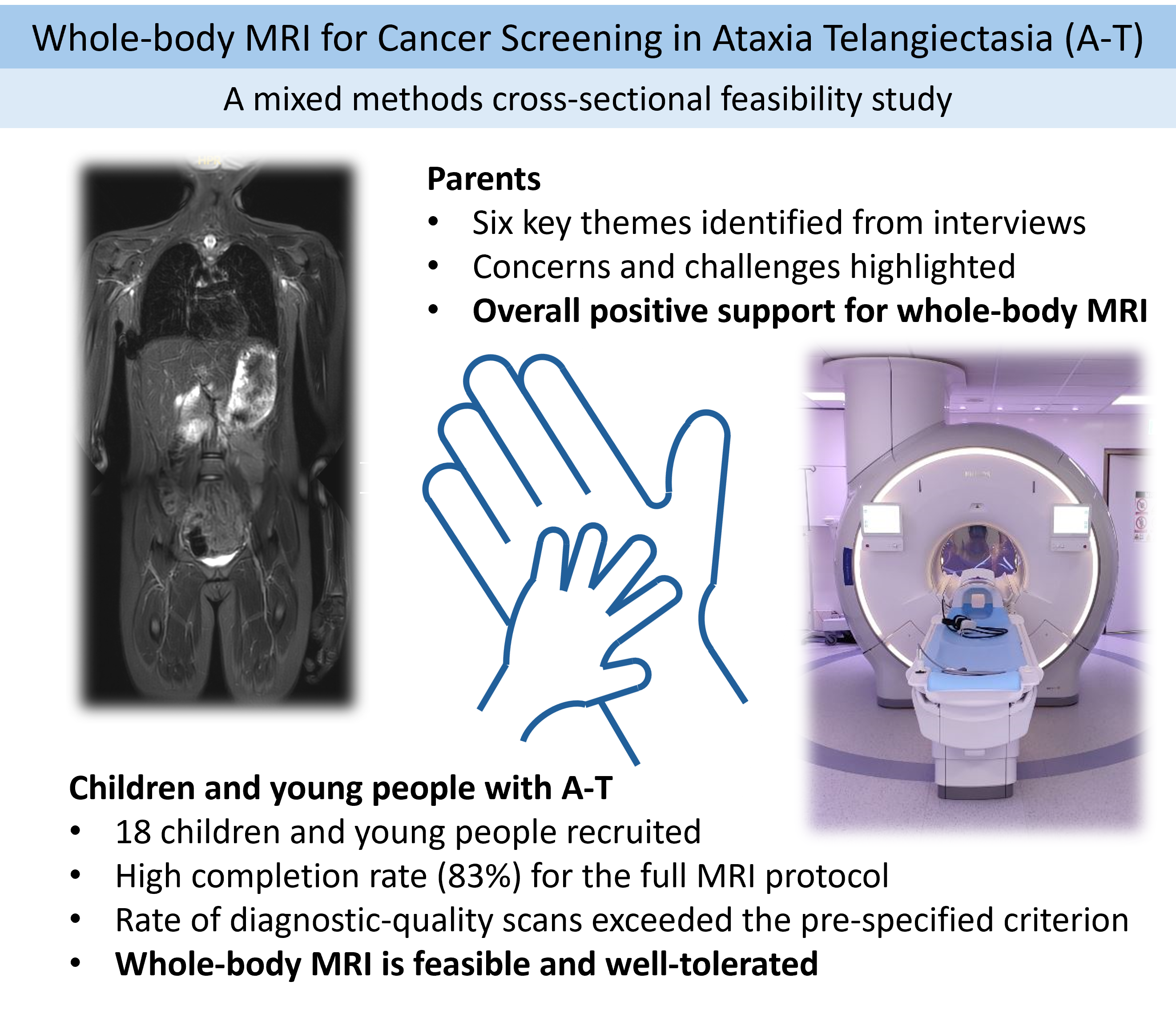

Supplement: Supplementary file 1 — Data S1: [file CAM4-13-e70049-s001.zip › WBMRIF~1.TIF]
